# SARS CoV-2 aerosol: How far it can travel to the lower airways?

**DOI:** 10.1063/5.0053351

**Published:** 2021-06-03

**Authors:** Mohammad S. Islam, Puchanee Larpruenrudee, Akshoy Ranjan Paul, Gunther Paul, Tevfik Gemci, Yuantong Gu, Suvash C. Saha

**Affiliations:** 1 School of Mechanical and Mechatronic Engineering, University of Technology Sydney (UTS), 15 Broadway, Ultimo, NSW 2007, Australia; 2 Department of Applied Mechanics, Motilal Nehru National Institute of Technology Allahabad, Prayagraj 211004, Uttar Pradesh, India; 3 James Cook University, Australian Institute of Tropical Health and Medicine, Townsville, QLD 4810, Australia; 4 Synergy CFD Consulting, Las Vegas, Nevada 89146, USA; 5 School of Mechanical, Medical and Process Engineering, Faculty of Engineering, Queensland University of Technology, Brisbane 4000, Australia

## Abstract

The recent outbreak of the SARS CoV-2 virus has had a significant effect on human respiratory health around the world. The contagious disease infected a large proportion of the world population, resulting in long-term health issues and an excessive mortality rate. The SARS CoV-2 virus can spread as small aerosols and enters the respiratory systems through the oral (nose or mouth) airway. The SARS CoV-2 particle transport to the mouth–throat and upper airways is analyzed by the available literature. Due to the tiny size, the virus can travel to the terminal airways of the respiratory system and form a severe health hazard. There is a gap in the understanding of the SARS CoV-2 particle transport to the terminal airways. The present study investigated the SARS CoV-2 virus particle transport and deposition to the terminal airways in a complex 17-generation lung model. This first-ever study demonstrates how far SARS CoV-2 particles can travel in the respiratory system. ANSYS Fluent solver was used to simulate the virus particle transport during sleep and light and heavy activity conditions. Numerical results demonstrate that a higher percentage of the virus particles are trapped at the upper airways when sleeping and in a light activity condition. More virus particles have lung contact in the right lung than the left lung. A comprehensive lobe specific deposition and deposition concentration study was performed. The results of this study provide a precise knowledge of the SARs CoV-2 particle transport to the lower branches and could help the lung health risk assessment system.

## INTRODUCTION

The thoracic cavity consists of inhalation and exhalation processes. During inhalation, airborne pollutants, for example, particulate matter, dust, smoke, pollens, viruses, or allergens, often in the form of liquid droplets and aerosols are ingested into the airways. Aerosol is a term first introduced in the 1920s and was initially used in the context of therapeutic inhalation (Anderson, 2005).

The inhaled air is ingested into the respiratory tract, commonly termed as human airways, which has a complicated geometry and hence is difficult to reconstruct even using computerized modeling. Due to the lack of computed tomography (CT)-scan images, earlier researchers (Weibel, 1963; Phillips and Kaye, 1995; Kitaoka *et al.*, 1999) used simplified (often with a regular cross-sectional shape) geometry of human airways. With the sophisticated imaging techniques, the researchers started developing more realistic geometry of human airways incorporating lung intricate shapes and minuscule anatomical features (Martonen *et al.*, 1995; Lizal *et al.*, 2012; Srivastav *et al.*, 2013; Frederix *et al.*, 2018) for conducting various computational studies. These studies include the effects of breathing through nasal and oral passages (Fitzpatrick *et al.*, 2003), transmission and deposition of inhaled aerosols, fine and ultrafine particles, etc., within both the upper and tracheobronchial airways (Fernández Tena and Casan Clarà, 2012; Mortazavi *et al.*, 2021). In the last two decades, respiratory fluid dynamics has matured enough to allow multiscale, multiphysics modeling (Burrowes *et al.*, 2008; Pozin, 2017) to analyze the various aspects of respiratory mechanics, starting from the inhalation mechanism (Islam *et al.*, 2020) to aerosol (Xi and Longest, 2007; Islam *et al.*, 2017; Lizal *et al.*, 2020) and drug delivery through pulmonary routes (Heyder, 2004; Kleinstreuer *et al.*, 2008) and even diseased airways (Martonen *et al.*, 2003; Srivastav *et al.*, 2014).

Refer to the term of aerosol, it is the combination between solid or liquid particles which are suspended in gas. Airborne transmission of many viral diseases is caused due to propagation of such airborne particles containing saliva, mucus, salts, cells, and even infectious pathogens-viral and/or bacterial particles (Wells, 1955). The droplets often originate from the viral infected inner epithelial layers of the respiratory tract surfaces (Mason, 2020) through exhalation, coughing, sneezing, talking, or vomiting by an infected person (Atkinson *et al.*, 2009). The ongoing COVID-19 pandemic has triggered the publication of numerous research articles encompassing various aspects and behavior of the SARS CoV-2 virus (Zhou and Zou, 2021). The virus mainly attacks human lung airways and eventually damages lung capacity of gas exchange (Mason, 2020). Hence, the study of the transport of SARS CoV-2 aerosol to the tracheobronchial airways (termed as lower airways) is important.

Prather *et al.* (2020) suggested that pulmonary infections are caused by small aerosols. Virus particles can suspend in air for a longer period and can be transmitted from an infected person to a non-infected person. Larger virus particles, on the other hand, can survive on the surfaces for a longer period and can be transmitted through contacts (Bhardwaj and Agrawal, 2020a, b). Kumar and Lee (2020) adopted continuous phase modeling for smaller aerosols, while discrete phase simulation was conducted for the larger aerosols. Diffusion equation-based Monte Carlo modeling was used by Vuorinen *et al.* (2020) for the transport of virus aerosols. Appropriate source and sink terms are added to the diffusion transport equations to represent the variable location of the infected people and source of ventilation, respectively. Xie *et al.* (2009) stressed the importance of including droplet distribution (i.e., variation in size) for discrete phase modeling since it is connected to the traveling path as well as the chances of viral infections (called viral load) in the case of SARS CoV-2. Liquid particle evaporation is another important phenomenon while transmitting the virus-laden aerosols, which is dependent on ambient temperature and saturation pressure. A recent study reported in the literature that small droplets released from the exhalation may be laden with COVID-19 virus, have very short evaporation timescale (≤1 s), and hence are evaporated as soon as it is ejected. Hence, the virus is considered as a particle (Chaudhuri *et al.*, 2020). The larger droplet or virus-laden particles are larger and cannot travel to the lower airways, as larger particles usually deposit to the upper airways. A recent study (Kwee and Kwee 2020) showed that nano-sized SARS CoV-2 Aerosol is deposited to various lobes of the lung in their radiographic images, which supports the assumption of this study. However, after inhalation the evaporation is largely regulated by the body temperature. Recently, a number of researchers (Feng *et al.*, 2020; Chaudhuri *et al.*, 2020; de Oliviera *et al.*, 2021) investigated various aspects of liquid particle evaporation and transmission in light of the COVID-19 pandemic. Smaller aerosols generated from continuous speech in a poorly ventilated room increased the infection risk by 11% as revealed in a recent study (de Oliviera *et al.*, 2021) and hence reiterates the importance of maintaining proper ventilation and physical distancing to avoid infection transmission.

With the outbreak of COVID-19 pandemic, a few researchers have also worked on the virus transmission in human airways. Madas *et al.* (2020) employed a lung model based on a stochastic deposition, which was developed by Koblinger and Hofmann (1990) to find out the deposition of viral loads and revealed that over 60% of the inhaled viral masses were deposited in the extrathoracalis (upper), which are the portion of the human lung airways, and suggested to affect the upper airways and if not diagnosed, could eventually develop into pneumonia. Other researchers focused on aerosol behavior in the intra-distal region of a simplistic lung model in the presence of different breathing conditions (Ciloglu, 2020), gravity and surface tension effects on micro-bubbles in simplistic bifurcated airways (Munir and Xu, 2020), mask-wearing effects in upper respiratory geometry (Xi *et al.*, 2020), aerosol transport in phantom lung bronchioles (Mallik *et al.*, 2020), cough exhalation from a 18-generation simplistic airways (Si *et al.*, 2021), etc.

The review of literature reveals a plethora of works on the transmission of infections and exhalation behavior originated from oral and nasal openings of the human airways. However, the transportation of virus-aerosols to the tracheobronchial human airways involving a realistic and detailed geometry of the airways has not yet been discussed and analyzed in detail. Since empirical evidence of SARS CoV-2 attacking the respiratory organ in the COVID-19 infected population exists, a Computational Fluid Dynamics (CFD) investigation of virus aerosol transport in a realistic human airways up to the 17th generation would help medical practitioners and inform further diagnosis and prognosis of the COVID-19 disease. The CFD studies of airflow and CoV-2 virus deposition in a digital reference model of the 17-generation airway were based on the anatomical model of an adult male, free of pathological alterations by Schmidt *et al.* (2004). The lung model consists of 1453 bronchi up to the 17th Horsfield order.

## NUMERICAL METHODS

The numerical study solved the air and particle transport equations and analyzed the particle flow in first 17-bifurcatins of the human lung. The Lagrangian scheme and Finite Volume based Discretization techniques are used for this study. The numerical calculation method in this study is performed and conducted based on ANSYS 19.2 (FLUENT). The governing equations are solved as follows:

$$\nabla .(\rho \vec{v})=0,$$ (1)

$$\nabla .(\rho \vec{v}\vec{v})=-\nabla p+\nabla .(\mu (\nabla \vec{v}+\nabla {\vec{v}}^{T}))+\rho \vec{g},$$ (2)

where static pressure is *p*, gravitational body force $\rho \vec{g}$, and molecular viscosity *μ*.

The internal energy equation is

$$\;\;\;\;\;\;\;\;\;\;\;\;\;\nabla \cdot \left(\rho \vec{v}e\right)\;=-\nabla \cdot \vec{J},$$ (3)

where e is the specific internal energy. The heat flux vector $\vec{J}$ is the sum of contributions due to heat conduction and enthalpy diffusion effects.

The inlet condition and velocity profiles are highly complex and irregular for person to person. No proper velocity profiles are established by the available literature. However, the flow inside the airway is similar to internal pipe flow and the flow become parabolic at the tracheal area of the airway. This study only considers the simulation, which starts from trachea to lower generations of the human lung as well as the trachea wall which is considered as the inlet of the airway. Therefore, a fully developed parabolic inlet condition (White, 2003) is used,

$$u(r)=2u_{av}(1-\frac{r^{2}}{R^{2}}),$$ (4)

where R is the pipe radius. The corresponding velocity for 7.5, 15, and 30 lpm cases is 0.4916, 0.9829, and 1.996 m/s, respectively, which is the maximum velocity. For a fully developed condition, maximum velocity is double of the average velocity.

SARS CoV-2 particles are smaller in size, and they are approximately around 120 nm. (https://www.pptaglobal.org/media-and-information/ppta-statements/1055–2019-novel-coronavirus-2019-ncov-and-plasma-protein-therapies).

Therefore, nano-particle transport equations are solved (Inthavong and Ahmadi, 2009),

$$\frac{du_{i}^{p}}{dt}=F_{D}+F_{\mathrm{Brownian}}+F_{\mathrm{Lift}}+\frac{\rho_{p}-\rho_{g}}{\rho_{p}}g_{i},\;F_{D}=\frac{1}{C_{c}}C_{D}A_{p}\frac{\rho_{g}\left|u_{i}^{g}-u_{i}^{p}\right|(u_{i}^{g}-u_{i}^{p})}{2m_{p}}=\frac{18\mu_{g}}{\rho_{p}d_{p}^{2}C_{c}}\left(u_{i}^{g}-u_{i}^{p}\right),\;C_{c}=1+\frac{2\lambda }{d_{p}}(1.257+0.4e^{\frac{-0.11d_{p}}{2\lambda }}),$$ (5)

where $F_{D}$ is the drag force per unit particle mass $m_{p}$, $C_{D}$ is the drag coefficient, $A_{p}$ is the cross sectional area of the particle, and $C_{c}$ is the Cunningham correction factor. λ is the mean free path of the gas molecules. $u_{i}^{p}$ is the i-th component of the time-averaged particle velocity while $u_{i}^{g}$ is the i-th component of the time-averaged gas (air) velocity. $\rho_{p}$ and $\rho_{g}$ are the density of particle material and gas (air), respectively. $g_{i}$ is the gravitational component. $\mu_{g}$ denotes the gas (air) viscosity and $d_{p}$ is defined as particle diameter. For the low particle Reynolds number ( Rep<0.5), the drag coefficient C_D_ can be defined as (Haider and Levenspiel, 1989)

$$C_{D}=\frac{24}{{Re}_{p}},\;{Re}_{p}<0.5.$$ (6)

The particle Reynolds number can be calculated from

$${Re}_{p}=\rho_{g}\frac{d_{p}\left|u_{r}\right|}{\mu_{g}},$$ (7)

where u_r_ is the relative velocity. The particle Re for 120 nm particle for this study is 0.0168.

Amplitude for Brownian force is applied as

$$F_{\mathrm{Brownian}}=\zeta \sqrt{\frac{\pi S_{0}}{\Delta t}},$$ (8)

where *ζ* is the unit variance for an independent Gaussian random number, time step integration of the particle *Δt*. The spectral intensity (*S*_0_) is defined as

$$S_{o}=\frac{216\mu k_{B}T}{\pi^{2}\rho_{p}d_{p}^{5}{\left(\frac{\rho_{p}}{\rho_{g}}\right)}^{2}C_{c}},$$ (9)

*T* is the fluid in absolute temperature, *k***_*B*_** is the Boltzmann constant, and *ρ_g_* is the gas density.

The SIMPLE coupling scheme and second order pressure discretization technique are employed. The second order upwind technique is utilized for energy and momentum equations. Hybrid initialization and pressure-based solver are used. The present model has considered the particle with a density of 1.0 g/cm^3^; all particles were initiated from the one inlet area that was the trachea. A steady injection method is used. In reality, SARS-CoV-2 particles are spherical in shape like other viruses (https://www.nih.gov/news-events/nih-research-matters/novel-coronavirus-structure-reveals-targets-vaccines-treatments). Goldsmith *et al.* (2020) reported that SARS CoV-2 viruses are spherical in shape, and the structure is surrounded by dark dots, which might have been interpreted as spikes on coronavirus. The transmission electron microscopy image also showed the spherical shape of the SARS CoV-2 virus. SARS CoV-2 aerosols are injected beginning from the inlet surface in the normal direction. The mass flow rate at the inlet is 0.5003 kg/s. The particles are injected from the inlet surface area, and each face of the surface injects a single particle. The particle distribution at the inlet surface is uniform, and all particles are injected at once. A total 14 800 particles were injected. The outlet boundary condition is used at the pressure outlet, and the open pressure condition is used at the terminal outlets.

The SARS CoV-2 aerosols were considered as the secondary phase, and the air was the continuous phase. The interaction between the discrete phase and continuous phase is considered. The maximum number of steps of tracking parameters is 5 × 10^−8^ and the step length factor is 5. Crowe *et al.* (2011) calculated the particle momentum response and collision time ratio, which eventually indicated whether the air was dilute or dense.

The momentum response time of the particle can be explained as

$$\tau_{V}=\frac{\rho_{p}d_{p}^{2}}{18\mu },$$ (10)

where particle density is *ρ_p_*, particle diameter is *d_p_*, and *μ* is the viscosity of air.

The time between the collisions can be defined as

$$\tau_{c}=\frac{1}{n\pi d_{p}^{2}v_{r}},$$ (11)

where n is the particle number and v_r_ is the particle relative velocity. If the ratio value is less than one, the fluid is dilute, and one-way coupling can be considered. The value obtained for the ratio in the present study was 0.000 41, which meant that this study is a one-way.

This study used “trap” boundary condition for the wall (Islam *et al.*, 2019). The physical meaning of the “trap condition” is particle will stick on the wall once it touches the wall. Once the particle touches the airway wall, the trajectory calculations will be terminated, and the fate of the particle will be recorded as “trapped.” The deposition fraction (DF) is defined as

$$\mathrm{DF}=\frac{\mathrm{Number}\;\mathrm{of}\;\mathrm{deposited}\;\mathrm{particles}\;\mathrm{in}\;\mathrm{the}\;\mathrm{wall}}{\mathrm{Number}\;\mathrm{of}\;\mathrm{virus}\;\mathrm{particles}\;\mathrm{entering}\;\mathrm{the}\;\mathrm{inlet}}.$$

## GRID REFINEMENT AND MODEL VALIDATION

**FIG. 1.**
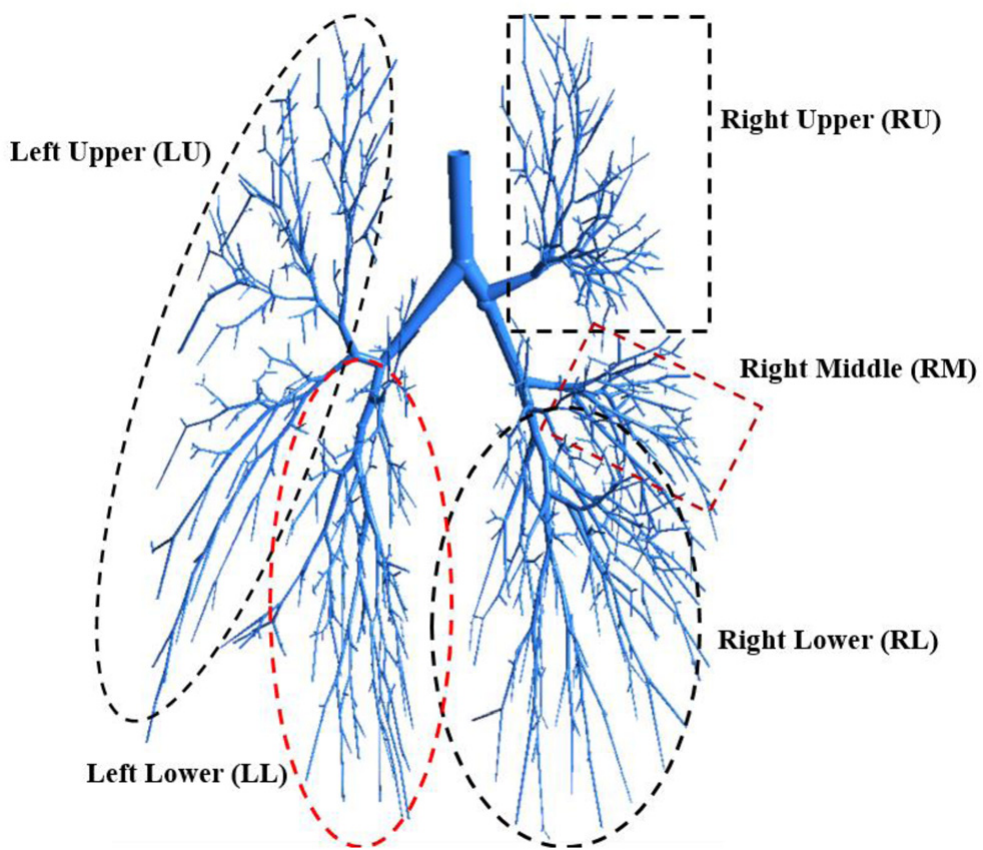
Highly asymmetric five-lobes 17-generation human lung model.

A 17-generation airway model (Gemci *et al.*, 2008; Schmidt *et al.*, 2004) is employed for the SARS CoV-2 aerosol transport and deposition to the lower part of the lung. Figure 1 shows the airway model with five different lobes. The study performed a proper grid refinement and the details of the mesh at different sections of the airway as well as the details of the grid refinement can be found in author's previous study (Islam *et al.*, 2018).

**FIG. 2.**
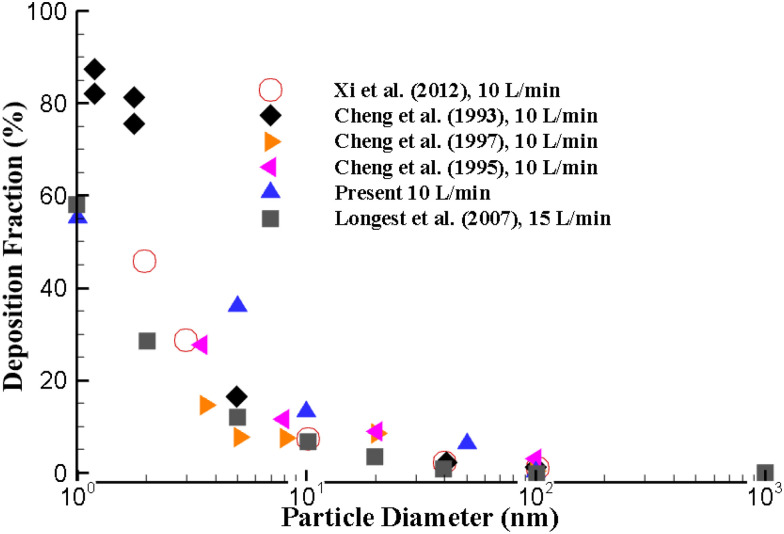
Numerical results validation with available literature at 10 l/min inlet airflow.

The numerical approach is validated with the available experimental and computations measurement in literature. A range of ultrafine particles are used to validate the deposition fraction (DF) at the upper airways. The ultrafine particle transport and DF are calculated for different airflow rates. Figure 2 illustrates the DF of the present calculation at 10 l/min inlet conditions with available literature data. The overall DF of the present study shows a close match with the available experimental and numerical measurements.

**FIG. 3.**
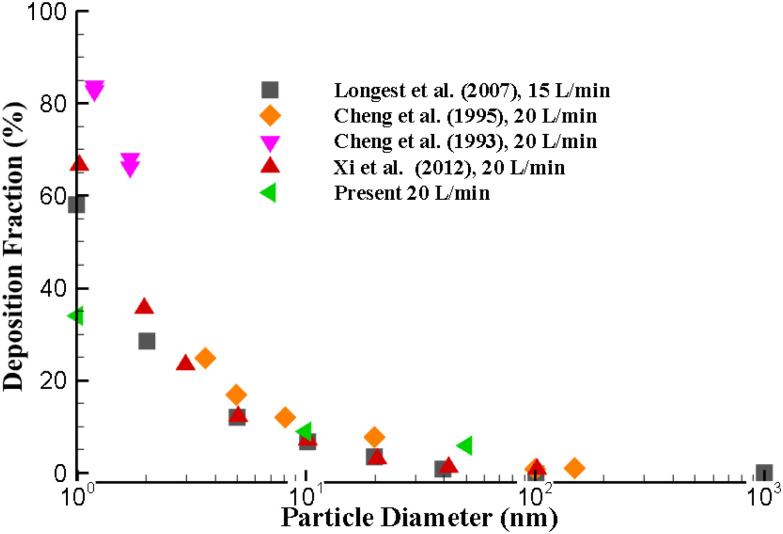
Numerical results validation with available literature at 20 l/min inlet flow (Cheng *et al.*, 1993, 1995; Longest and Xi, 2007; Xi *et al.*, 2012).

The DF of the present approach at higher flow rate is also compared with the existing literature (Cheng *et al.*, 1993, 1995, 1996; Longest and Xi, 2007; Xi *et al.*, 2012). Figure 3 demonstrates the comparison of DF at 20 l/min flow rate at the upper airways. The DF for the smaller diameter particle is found to be higher when compared to the larger diameter nano-particle, which also support the hypothesis of the Brownian motion. The DF of this present calculation indicates a good agreement with the available results for larger nano-size particles.

## RESULTS AND DISCUSSION

The airflow and SARS CoV-2 aerosol transport to the lower part of lung are simulated for different inlet conditions. A highly asymmetric 17-generation bifurcating model is utilized to analyze the SARS CoV-2 aerosol transport and deposition to the lower airways. The overall investigation is performed for three different airflow rates, which consist of 7.5, 15, and 30 l/min.

**FIG. 4.**
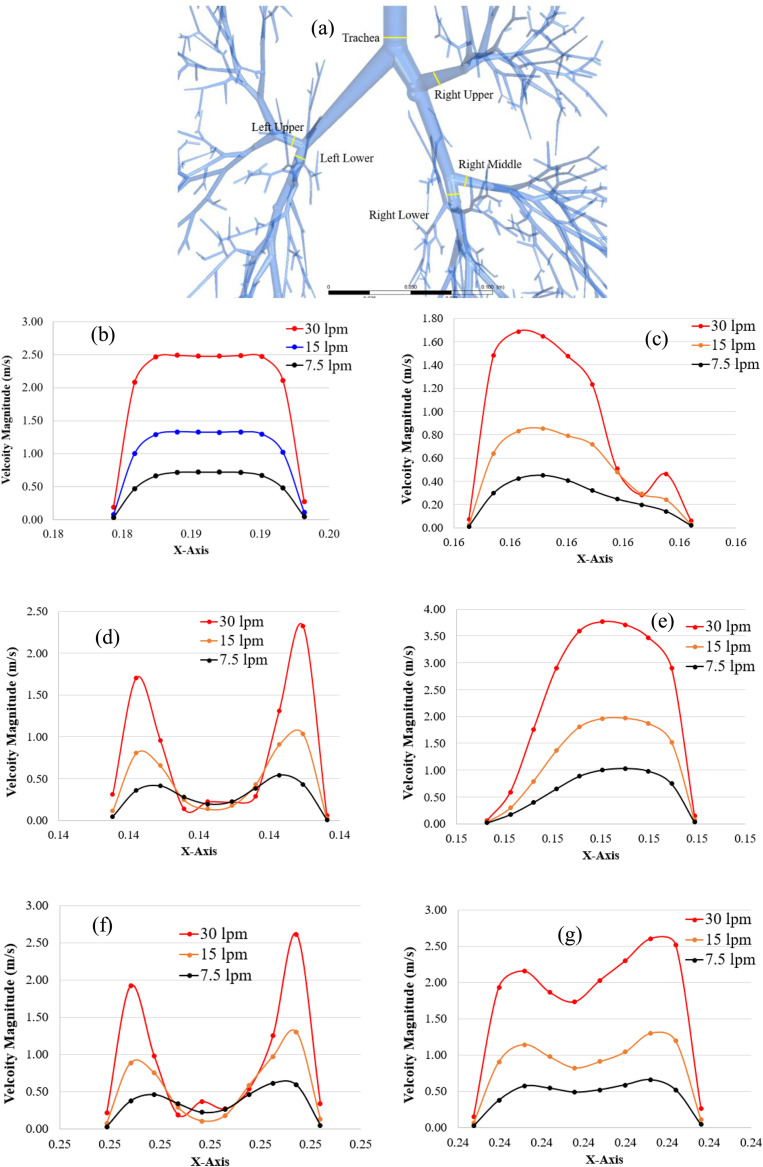
Air velocity profiles at the randomly chosen location of the airway, (a) random location definition, (b) trachea, (c) right upper (RU) lobe, (d) right middle (RM) lobe, (e) right lower (RL) lobe, (f) left upper (LU) lobe, and (g) left lower (LL) lobe.

The velocity profiles are plotted at various positions of the upper airways, and the lungs at the left side and right side for different breathing conditions. SARS CoV-2 aerosol usually follows the air streamline inside the respiratory tube. An accurate understanding of the upper and lower airways flow pattern is important to analyze the SARS CoV-2 transport and lung deposition. Figure 4 presents the velocity profiles for three different flow rates at selected cross-sections in different areas for the 17-generation lung model, from the trachea to terminal part of the lung. Figure 4(a) presents the velocity profiles at the trachea. Figures 4(b)–4(d) present the velocity profiles at three lobes (upper, lower, and middle) of the right lung. Figures 4(e) and 4(f) present the velocity profiles at two lobes (lower and upper) of the left lung.

Figure 4(a) shows the randomly selected locations at trachea and various lobes of the airway model. The velocity profiles for all three airflow rates at the tracheal area show a fully developed behavior [Fig. 4(b)] and the velocity magnitude is maximum at the center of the airways for all cases. However, the velocity field for all lobes tends to be locally transitional, especially at the RM lobe [Fig. 4(d)] and LU lobe [Fig. 4(f)], which have similar air velocity magnitudes and nearly reach the 0.1 m/s at the middle point of the cross section for all three airflow rates. The velocity magnitude is higher close to the airway wall for RM and LU lobes, which potentially increases the SARS CoV-2 aerosol deposition at the airways of the RM and LU lobes. At the RU lobe, the velocity profile for 30 l/min shows a different trend compared to other flow rates [Fig. 4(c)]. At 30 l/min condition, the flow becomes locally unstable. The velocity magnitude at the RL lobe [Fig. 4(e)] is found maximum, whereas the RU lobe [Fig. 4(c)] is found to have the lowest velocity magnitude.

**FIG. 5.**
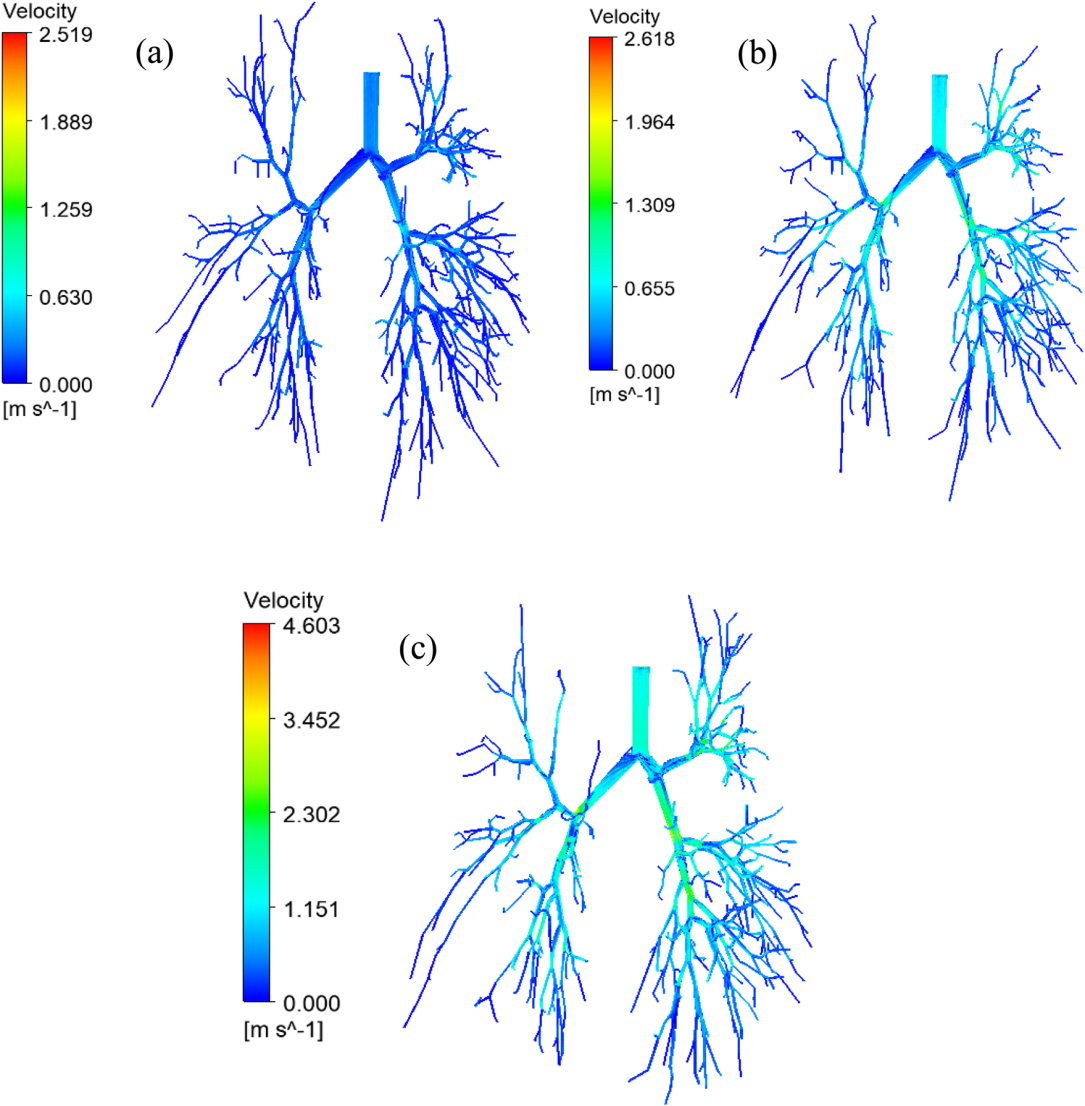
Velocity streamline at different airflow rates, (a) 7.5, (b) 15, and (c) 30 l/min.

Figure 5 presents the velocity streamlines throughout the bifurcating model for various airflow rates. Figure 5(a) shows the air velocity streamlines at 7.5 l/min, whereas Figs. 5(b) and 5(c) present the air velocity streamline at 15 and 30 l/min, respectively. The overall velocity streamline shows a higher velocity magnitude at the upper area of the bifurcating model. The velocity streamline figure for low inlet velocity conditions [Fig. 5(a)] indicates relatively low-velocity magnitude to the terminal bronchioles than the high-velocity conditions [Fig. 5(c)]. For the low inlet flow condition [Fig. 5(a)], the highest velocity magnitude is reported at the upper bronchioles for all three right lobes. On the contrary, 15 l/min [Fig. 5(b)] and 30 l/min [Fig. 5(c)] have the highest air velocity at the initial area for all five main lobes.

**FIG. 6.**
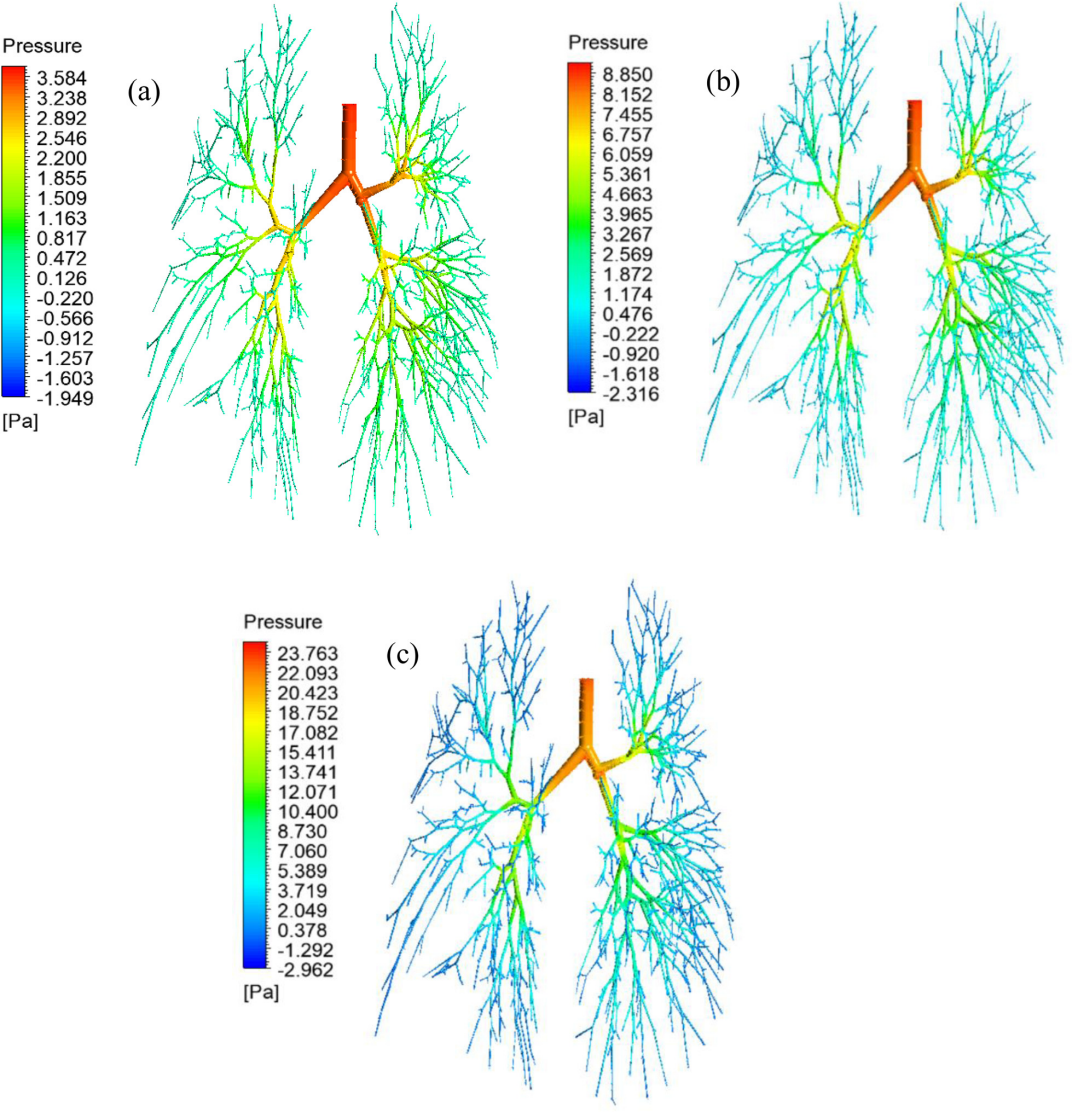
Pressure contour throughout the lung airways, (a) 7.5, (b) 15, and (c) 30 l/min.

The precise knowledge of the airway pressure and pressure-drop to the terminal airways is important for airway health risk analysis. Figure 6 presents the pressure contours for all three different airflow rates that include 7.5 l/min [Fig. 6(a)], 15 l/min [Fig. 6(b)], and 30 l/min [Fig. 6(c)]. Figure 6 reports that the pressure generally decreases from the initial area (trachea) to the lower generation (17th generation). The pressure at the tracheal wall and the upper airways is found maximum for all cases, and a significant pressure drop is obviously found in the lower airways. The maximum pressure of 23.763 Pa is found at the highest airflow rate at 30 l/min [Fig. 6(c)], while the maximum pressure of 3.584 Pa is found at the lowest airflow rate at 7.5 l/min [Fig. 6(a)]. Figure 6(a) shows, at the low inlet condition (7.5 l/min), the pressure drop from the upper airways to the lower airways is insignificant. At the high inlet velocity condition (30 l/min), the pressure drop is found significant from the upper airways to the lower airways. At high flow conditions, the terminal airways velocity magnitude is found relatively higher than the low inlet condition, which is reported in Fig. 5(c). The higher velocity magnitude at the terminal airways eventually generates low pressure at the lower airways.

**FIG. 7.**
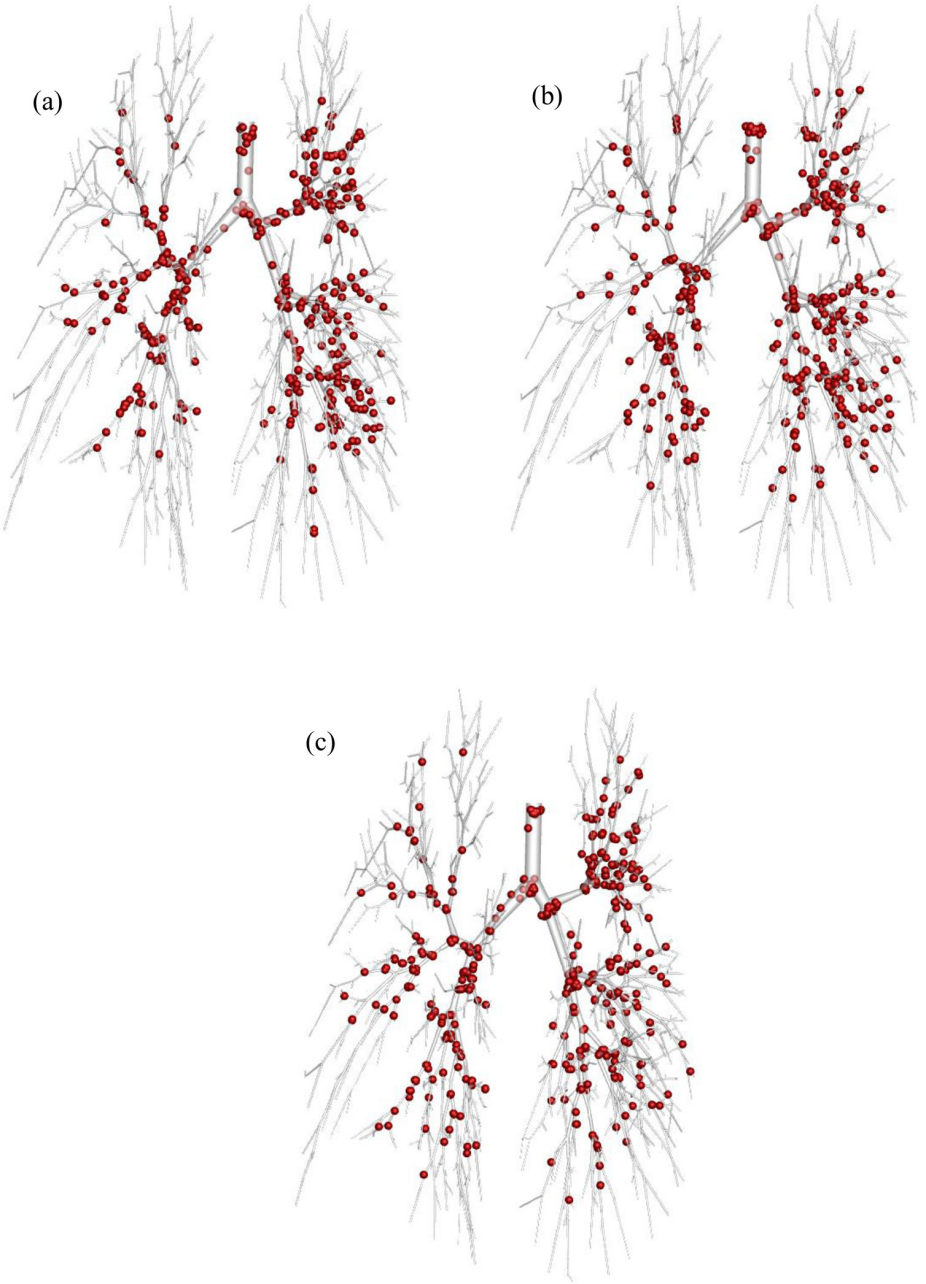
SARS CoV-2 Aerosol deposition (120 nm) at different physical conditions (a) 7.5, (b) 15, and (c) 30 l/min flow rate. (Sphere size is increased during post-processing for visualization purpose.)

The SARS CoV-2 Aerosol deposition scenario is presented in Fig. 7 under various airflow rate conditions. Figure 7(a) illustrates the deposition for airflow rate at 7.5 l/min, while Figs. 7(b) and 7(c) shows the deposition for the airflow rate at 15 and 30 l/min, respectively.

Figure 7 shows that the SARS CoV-2 aerosols are more trapped in the tracheal area and the bifurcation of all generations in the lung lobes for both lung sides. Figure 7 shows the higher SARS CoV-2 aerosol which is trapped at the tracheal inlet, and upper airways at 7.5 l/min [Fig. 7(a)] and 15 l/min [Fig. 7(b)] compared to the airflow rate at 30 l/min [Fig. 7(c)]. The Brownian motion effect is dominant for smaller aerosol like SARS CoV-2 transport and deposition. SARS CoV-2 aerosol can spontaneously transport through the airways at a low flow rate, and the random movement of the SARS CoV-2 aerosol increases the overall deposition rate at the upper airways. Simultaneously, the Brownian motion effect becomes less effective with the increase in the velocity magnitude. The SARS CoV-2 aerosol deposition at the right side is higher than the left side for all airflow rates. At 15 l/min [Fig. 7(b)] and 7.5 l/min [Fig. 7(a)] shows a cluster of SARS CoV-2 aerosol deposition at the three right lobes, whereas the high airflow rate at 30 l/min [Fig. 7(c)] shows a cluster SARS CoV-2 aerosol deposition at the upper area of the right side only.

**FIG. 8.**
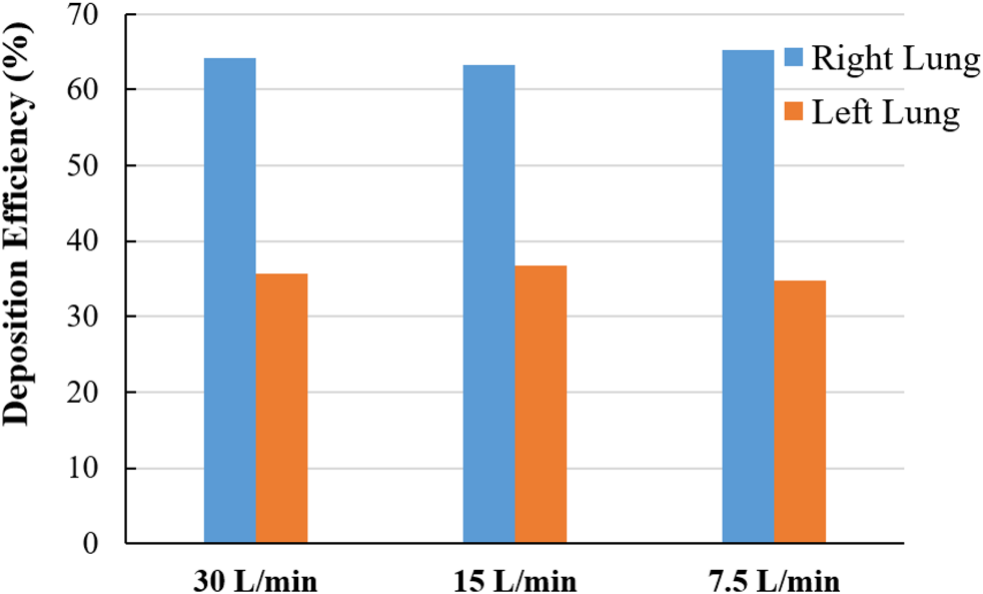
DE comparison at right and left lung for different airflow rate conditions.

Comparing SARS CoV-2 aerosol deposition efficiency (DE) between the right and the left lung is calculated, and Fig. 8 reports the DE under the various airflow rate conditions. Figure 8 shows that the DE of the SARS CoV-2 aerosol at the left side is found to be lower when compared to the right side for all airflow rates. At 7.5 l/min, the highest DE 65.22% is reported at the right lung, and the highest DE at the right lung causes the lowest DE at the left lung. At the 30 l/min inlet case, the DE of the SARS CoV-2 aerosol in the lung at the right side is 64.25% and the left side is 35.75%. The anatomical structures of the right lung and left lung are different, and the right lung airway diameter is higher than that of the left lung. A number of studies analyzed the total flow distribution (%) in the right lung and left lung and found higher flow distribution to the right lung than the left lung. Cohen *et al.* (1990) found 60% of the total flow goes through the right lung, Horsfield *et al.* (1971) reported 54.6% of the total flow goes through the right lung, and Islam *et al.* (2018) reported 54.93% total flow goes through the right lung. Higher flow distribution to the right lung indicates a higher amount of particle will go through the right bronchioles, which increases the deposition efficiency at the right lung. The SARS CoV-2 aerosol follows the air pathlines and more SARS CoV-2 aerosol enters the right bifurcations due to high flow distribution to the right lung. The higher SARS CoV-2 aerosol in the right bifurcations increase the overall DE at the right lung.

**FIG. 9.**
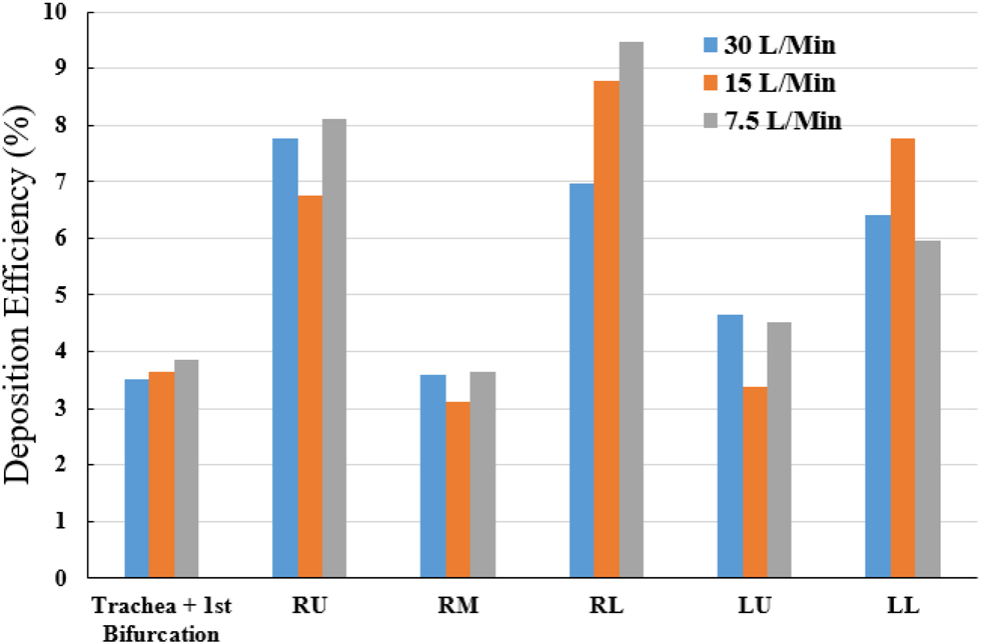
Local deposition of SARS CoV-2 aerosol at different inlet conditions. LU, left upper lobe; LL, left lower lobe; RU, right upper lobe; RM, right middle lobe; and RL, right lower lobe.

**FIG. 10.**
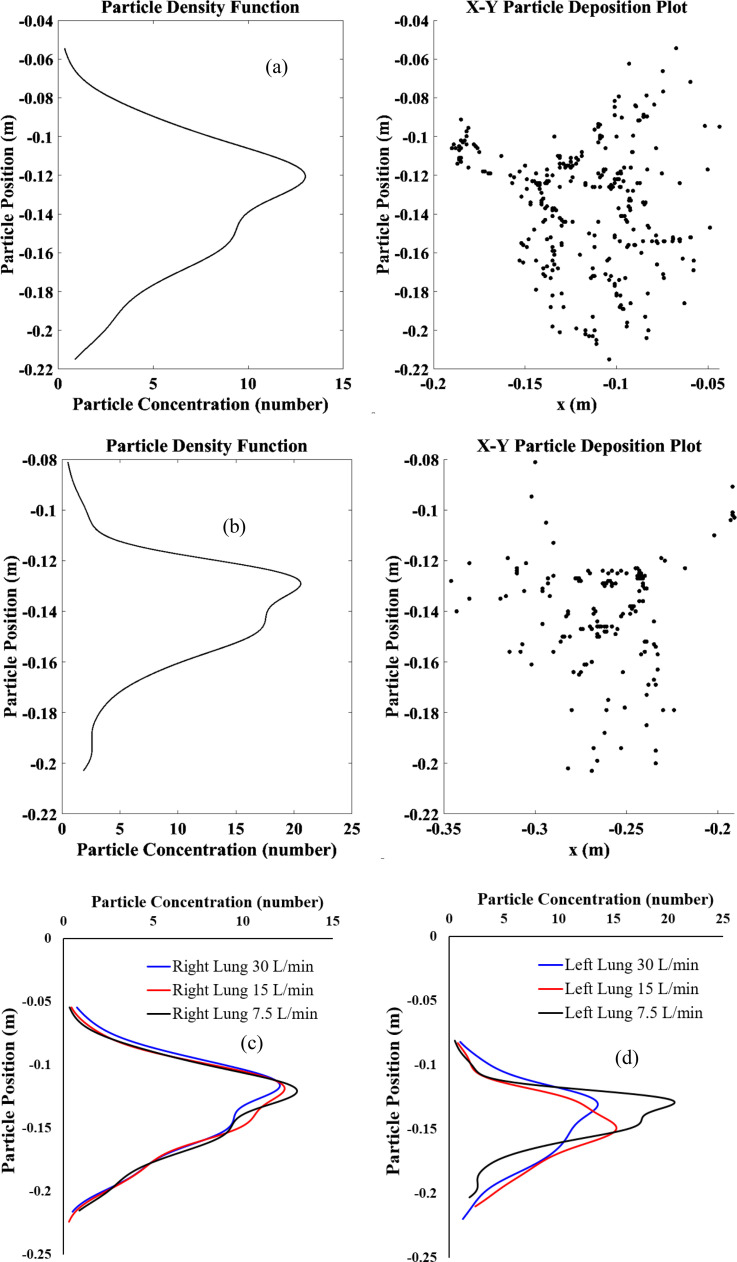
SARS CoV-2 aerosol deposition concentration from trachea to the terminal airways for various airflow rate conditions, (a) 7.5 l/min at right lung, (b) 7.5 l/min at left lung, (c) right lung, and (d) left lung.

Figure 9 presents the SARS CoV-2 aerosol deposition at local areas involving the trachea and first bifurcation, the three lung lobes of the right side, and the two lung lobes of the left side. The local DE of the SARS CoV-2 aerosol is calculated for different airflow rates. The local DE of the SARS CoV-2 aerosol illustrates higher deposition at the trachea and first bifurcation region at low inlet condition (7.5 l/min). At the 7.5 l/min condition, 3.85% of the total SARS CoV-2 aerosols are deposited at trachea and first bifurcation area, whereas 3.51% SARS CoV-2 aerosols are deposited for 30 l/min case. The local SARS CoV-2 aerosol DE at the bronchioles of the RL lobe is found higher than other lobes. At the 7.5 l/min inlet condition, 9.46% of the total SARS CoV-2 aerosols are deposited at the RL lobe, whereas 6.96% for 30 l/min airflow rate. At the 15 l/min inlet case, the SARS CoV-2 aerosol deposition DE is found maximum at the LL lower lobe which is 7.77%. The overall SARS CoV-2 aerosol DE curve reports that the DE for 30 l/min inlet case is lower at all lobes, including the trachea and first bifurcation of the 17th generation model. For the comparison between the two lung sides, the overall DE at the right side is found to be higher than the left side. The DE at the RM lobe and LU lobe is lower than other areas. To be concluded, the SARS CoV-2 aerosols are mostly trapped at the RL lobe and RU lobe and rarely trapped at the RM lobe and LU lobe for 7.5 and 30 l/min. In contrast, for 15 l/min, the majority of the SARS CoV-2 aerosol deposition generally locates at RL lobe and LL lobe, while the minority of this aerosol deposition is in the RM lobe and LU lobe. The first-ever lob-specific SARS CoV-2, aerosol DE analysis for the 17-generation model, would improve the knowledge of the SARS CoV-2 transport to the lower part of the lung airways of a large-scale model. A recent study has investigated the (Kwee and Kwee, 2020) CT-images of SARS CoV-2 positive patient from a Reverse Transcription (RT)-Polymerase Chain Reaction (PCR) test. The CT-Scan image reports the SARS CoV-2 presence at the RU lobe [Fig. 10(a)], RM and LL lobe [Fig. 10(b)], RU lobe [Fig. 10(c)], and both lower lobes [Fig. 10(d)], which necessarily indicate the significance of the present study. A comprehensive lob-specific analysis is presented in Fig. 9, which would potentially improve the knowledge of the field.

Figure 10 demonstrates the SARS CoV-2 aerosol deposition concentration in the right lung and left lung in the different airflow rate conditions. This aerosol deposition concentration is plotted based on the SARS CoV-2 aerosols x, y, z in the airway wall. Figure 10(a) presents the SARS CoV-2 aerosol concentration for 7.5 l/min flow condition at the right lung, while Fig. 10(b) shows the left lung concentration. Both figure's right panel shows the deposited SARS CoV-2 aerosol and the left panel shows the deposition concentration curve. The concentration curve on the left panel demonstrates the deposition hot spot (DHS) at the bifurcating airways that is presented at the right panel of the figure. The concentration curve shows the SARS CoV-2 aerosol deposition concentration is found to be higher at the upper as well as middle bifurcations of the 17-generation model. A comprehensive SARS CoV-2 aerosol deposition concentration for all cases is presented in Figs. 10(c) and 10(d). At right lung, the SARS CoV-2 aerosol concentration shows a similar trend for all cases whereas a different trend is observed at the left lung. The asymmetric brunching pattern of the left and the right lung influences the overall SARS CoV-2 aerosol transport to the lower airways. This analysis would provide an understanding of the SARS CoV-2 aerosol deposition hot spot at the lower airways of a large model.

## CONCLUSIONS

In this paper, the SARS CoV-2 aerosol transport to the lower part of the lung airways of a 17-generations lung airway model is investigated numerically for the first time. The SARS CoV-2 aerosol transportation to the lower airways is investigated for different inlet conditions. The key findings of the study are listed as follows:

• The cluster of SARS CoV-2 aerosols is found at the right lung, which is more than one time of the left lung for all airflow rates. A total of 35.55%, 33.45%, and 32.90% of SARS CoV-2 injected aerosols are deposited to the airway wall for 7.5, 15, and 30 l/min, respectively. The remaining aerosols escape and transport to lower generations and alveolar region.
• The highest deposition efficiency is located at the RL lobe with the low airflow rate of 7.5 l/min, whereas the lowest DE is found at the RM and LU lobes with the airflow rate of 15 l/min.
• The majority of SARS CoV-2 aerosols is trapped at RL and RU lobes, and the minority is trapped at RM and LU lobes for 7.5 and 30 l/min airflow rates. For 15 l/min, the minority of aerosol deposition is in the RM and LU lobes which are similar to other airflow rates but the majority of this aerosol deposition is located at RL and LL lobes instead.
• The SARS CoV-2 deposition concentration curves show a similar trend for the right lung, while the left lung is different. The deposition hot spot (DHS) of the right lung is found at the first bifurcation of the RU lobe. For the left lung, the DHS is found at LU and LL lobes for 7.5 and 30 l/min, while the 15 l/min has the DHS point at LU lobe only.

The numerical study demonstrates the SARS CoV-2 aerosol transport and deposition concentration at different lobes of the large airway model. The numerical study investigated the SARS CoV-2 aerosol transport to the lower area of the lung airways of a 17-generation model for the first time and a comprehensive lobe-specific analysis is performed, which would improve the SARS CoV-2 aerosol transport knowledge to the lower airways and help the health risk assessment of the covid patients. The numerical study also analyzed the deposition hotspot of the SARS CoV-2 aerosol to the right and the left lung. The present study along with a more patient-specific study would improve the knowledge of the field. The future study will investigate the age and patient-specific whole lung model for better understanding of the SARS CoV-2 aerosol to the lower airways.

### Assumptions of the study

In reality, the aerosol emitted during exhalation exhibits a wide size distribution. The smaller droplet could evaporate during transportation and become smaller. During exhalation, the aerosol could contain a single SARS CoV-2 virus or more than one SARS CoV-2 virus. If the aerosol contains more than one SARS CoV-2 viruses, then the size and shape of the virus-laden particle could be different. This study assumed a single isolated virus and did not consider the aggregation of the SARS CoV-2 viruses. The future study will perform a comprehensive analysis on virus-laden particles, and the aggregation of the viruses on aged people lung as the virus is found deadly for older people. The study assumed that virus particles have no electrical charges for the intermolecular forces and van der Waals interactions are neglected as the study did not investigate the particle and lung surfactant interaction.

## Data Availability

The data that support the findings of this study are available from the corresponding authors upon reasonable request.
